# Retrospective review of Andexanet Alfa versus 4-Factor Prothrombin Complex Concentrate for reversal of DOAC-Associated Intracranial Hemorrhage

**DOI:** 10.1007/s11239-022-02715-4

**Published:** 2022-11-10

**Authors:** Camille Troyer, Wesley Nguyen, Annie Xie, Dexter Wimer

**Affiliations:** Department of Clinical Pharmacy, UCSF Medical Center, San Francisco, USA

## Abstract

**Background**: Mortality of oral anticoagulation-associated ICH is around 60%, with oral anticoagulation increasing the risk of ICH seven to ten-fold compared to no anticoagulation. Current guidelines recommend DOACs (direct oral anticoagulants) as first-line therapy in the treatment of VTE (Venous Thromboembolism) due to their more favorable safety profile. There are two agents available for DOAC reversal, Coagulation Factor Xa (recombinant), inactivated-zhzo (andexanet alfa, Andexxa®) and 4-factor prothrombin complex concentrate (4FPCC). There is little data comparing the two agents in real-life clinical settings.

**Objective**: The primary objective of this study was to determine if there was a difference in hemostatic efficacy of andexanet alfa and 4FPCC in patients with a factor Xa inhibitor-related intracranial hemorrhage.

**Methods**: This was a retrospective, single-center study conducted in adult patients admitted at a quaternary academic medical center from September 2017 to March 2021. Adults with a diagnosis of intracranial hemorrhage (ICH) were included if they received either 4FPCC or andexanet alfa for reversal of apixaban or rivaroxaban. In addition to hemostatic efficacy per imaging, we assessed disposition location, cerebral performance score, blood product consumption, and the development of a new thrombus.

**Results**: A total of 46 patients were included in this study, 15 received 4FPCC (32%) and 31 received andexanet alfa (68%). There was no difference in the proportion of patients with excellent (4FPCC 9 [60%] vs. andexanet alfa 16 [51.6%], p = 0.61), good (4FPCC 2 [13.3%] vs. andexanet alfa 7 [22.6%]), or poor (4FPCC 1 [6.7%] vs. andexanet alfa 5 [16.1%]) hemostasis after administration of each agent. There were no significant differences in any secondary outcomes.

**Conclusion and Relevance**: Our study found no difference in hemostatic efficacy between andexanet alfa and 4FPCC. At this time, clinicians should choose an agent based on individual patient presentation and resource availability. Further research will help clarify the role of each agent in the management of DOAC-related intracranial hemorrhage.

## Introduction

On average, thrombosis results in one death every six minutes in the United States [1].Thrombosis in blood vessels can interfere with flow in the extremities, resulting in deep vein thrombosis (DVT). Without intervention, the thrombus can break off and travel through the bloodstream to the lungs, resulting in pulmonary embolism (PE). Collectively, DVT and PE are known as venous thromboembolism (VTE) and result in over 100,000 deaths per year [1]. Rather than thrombolysis, the American Society of Hematology (ASH) 2020 guidelines prefer anticoagulation for treatment of VTE [2]. However, all anticoagulation therapies are associated with a risk of major bleeding, with one of the highest mortality and morbidity complications being intracranial hemorrhages (ICH). Mortality of oral anticoagulation-associated ICH is about 60%, and oral anticoagulation increases the risk of ICH seven to ten-fold compared to no anticoagulation [3]. While all anticoagulation therapies carry a risk of major bleeding, direct oral anticoagulants (DOACs) are reported to have a 50% reduction in ICH risk compared to Vitamin-K Antagonist (VKA) therapy [4]. Current guidelines recommend DOACs as first-line therapy in the treatment of VTE due to their more favorable safety profile [5].

Currently, there are two agents available for DOAC reversal, Coagulation Factor Xa (recombinant), inactivated-zhzo (andexanet alfa, Andexxa®) and 4-factor prothrombin complex concentrate (4FPCC). 4FPCC was FDA approved in 2013 for the reversal of acute acquired coagulation factor deficiency induced by VKA therapy [6]. Since its approval, 4FPCC has been the standard of care for the reversal of VKA-associated bleeding. While only FDA indicated for reversal of VKA therapy, 4FPCC has also been utilized off-label in the setting of life-threatening factor Xa inhibitor-associated hemorrhages. In 2018, the FDA approved andexanet alfa as the first reversal agent for patients on factor Xa inhibitor therapy. In contrast to 4FPCC, which contains heparin inactivated coagulation factors II, VII, IX, and X, as well as antithrombotic human proteins C and S, andexanet alfa is a recombinant decoy factor Xa that binds specifically to rivaroxaban and apixaban. Andexanet alfa has a higher affinity to these medications than innate factor Xa, which negates their anticoagulant effect [7]. As such, 4FPCC can be conceptualized as a broad-spectrum hemostatic agent, while andexanet alfa is a targeted therapy and is only indicated in reversal of the effects of rivaroxaban and apixaban. While 4FPCC has been used off-label for reversal of DOACs, the safety and efficacy of andexanet alfa in the treatment of major bleeding in patients treated with other factor Xa inhibitors, such as edoxaban, [8] is not indicated at this time due to questions of safety and efficacy. The objective of this study was to retrospectively evaluate if there was a difference in hemostatic efficacy between andexanet alfa and 4FPCC in patients with a factor Xa related ICH at a quaternary academic medical center.

Dosing of andexanet alfa is based on dose and time since last dose of rivaroxaban or apixaban as seen in Table 1. 4FPCC most commonly utilizes a standardized weight-based dosing of 50 units per kilogram up to 5000 units for DOAC reversal. There is also a significant difference in cost between the two reversal agents, with high dose andexanet alfa costing roughly five times the average wholesale price of 4FPCC (Table 2). Currently, there is a lack of prospective randomized head-to head studies comparing the safety and efficacy of andexanet alfa versus 4FPCC for factor Xa inhibitor-associated ICH. Ammar et al’s 2021 retrospective, single-center study did not find significant differences in functional outcomes when comparing patients treated with 4FPCC and andexanet alfa for apixaban or rivaroxaban related ICH [9]. However, the small sample size and limited statistical power make external validity and application challenging. Additionally, Nederpelt et al’s 2021 systematic review and meta-analysis concluded that current studies do not unequivocally support the clinical superiority of andexanet alfa over 4FPCC in the reversal of factor Xa inhibitor-associated acute major bleeding. 10 Further studies are needed to determine whether there are significant differences in clinical and functional outcomes between patients with apixaban or rivaroxaban related ICH treated with 4FPCC or andexanet alfa.

**Table 1:** Andexanet alfa Dosing

Direct Factor Xa Inhibitor	Last dose	Last factor Xa inhibitor < 8 h or Unknown	Last factor Xa inhibitor ≥ 8 h
Apixaban	≤ 5 mg	Low dose	Low dose
Apixaban	> 5 mg/unknown	High dose	Low dose
Rivaroxaban	≤ 10 mg	Low dose	Low dose
Rivaroxaban	> 10 mg/unknown	High dose	Low dose

*Low dose: 400 mg IV bolus, followed by 4 mg/min IV infusion for up to 120 min (480 mg)*

*High dose: 800 mg IV bolus, followed by 8 mg/min IV infusion for up to 120 min (960 mg)*

**Table 2:** Cost comparison of andexanet alfa vs. 4FPCC

Treatment Agent	Dose	Cost*
Andexanet alfa	High Dose	$ 46,406
	Low Dose	$ 25,781
4FPCC	Max dose (5000 units)	$ 9,665

*Cost based on institutional wholesale acquisition cost

## Methods

This was a retrospective, single-center study conducted in adult patients admitted to our institution from September 2017 to March 2021. Our institution is a 600-bed quaternary care academic medical center. Patients 18 years of age or older with a diagnosis of intracranial hemorrhage (ICH) were included in the study if they received either 4FPCC or andexanet alfa for reversal of apixaban or rivaroxaban. Patients were excluded if they received andexanet alfa or 4FPCC for an indication other than ICH. Prior to the FDA approval of andexanet alfa in May of 2018, UCSF patients received 4FPCC via rapid IV infusion at a dose of 50 units/kg up to 5000 units with a maximum infusion rate of 8.4 mL/min. Andexanet alfa dosing was dependent on the dose of the factor Xa inhibitor and when the timing of the patient’s last dose as seen in Table 1. This study was IRB approved at our institution. The electronic medical record (EPIC, Verona, WI) was reviewed to record patient demographic information for all study participants. All data was previously obtained in the course of standard patient care. Study data were collected and managed using REDCap electronic data capture tools [11, 12].

The primary outcome of this study was the hemostatic efficacy of andexanet alfa and 4FPCC in patients with a factor Xa inhibitor-related intracranial hemorrhage. Hemostatic efficacy was identified as excellent, good, poor, or not reported according to CT scan readings after administration of either 4FPCC or andexanet alfa (Table 3). The classification categories are similar to those outlined in the ANNEXA-4 study. All patient CT scans were formally read and interpreted by radiologists as per institutional standard with no official time interval pre-determined, but at physician discretion. Secondary clinical and safety outcomes included time from order entry to administration of treatment, thrombotic event within 30 days, survival to discharge, and discharge location. Discharge locations for patients were home without assistance, home with nursing care, or skilled nursing facilities. Cerebral performance category (CPC) scores were also collected at discharge. CPC scores feature categories 1 (good cerebral performance) to 5 (brain death). Generally, CPC scores of 1 to 2 are considered favorable and CPC scores of 3 to 5 are considered unfavorable in terms of patient specific neurological function and outcomes.

**Table 3:** Hemostatic Efficacy Classification

	Excellent	Good	Poor
CT impression post-intervention	No new bleed or increase in hematoma volume	Slight increase in hematoma volume	New bleed or significant increase in hematoma volume

All data was analyzed using the appropriate statistical test for normally and non-normally distributed data as follows. For continuous normally distributed data we used the t-test, and for non-normally distributed data we used the Wilcoxon rank-sum test. For non-continuous data we used the chi square or Fisher’s exact as observation size dictated. All analyses were performed using R software (R, version 4.1.2, R Core Team (2021). R: A language and environment for statistical computing. R Foundation for Statistical Computing, Vienna, Austria). Skewed continuous data is reported as median with IQR whereas normally distributed data is reported as mean with standard deviation. Based on available published data using andexanet alfa, there was an expectation that 25% of patients may have a poor outcome, assuming a 3:1 distribution between the andexanet alfa and 4FPCC groups, respectively, 77 patients were needed in this study to meet statistical power, 50 in the andexanet alfa group and 27 in the 4FPCC. 15.

**Table 4:**
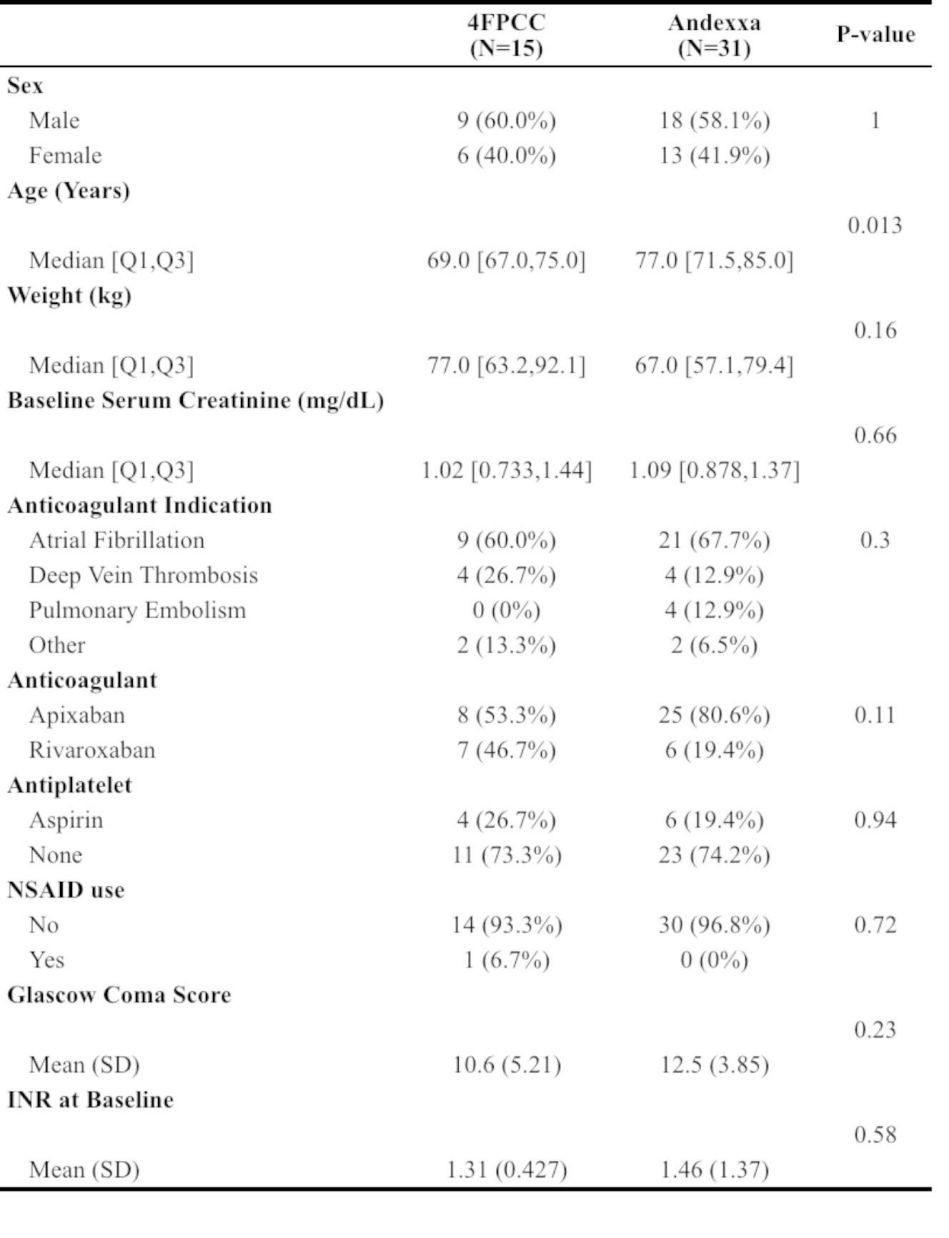
Baseline characteristics of all included patients

**Table 5:**
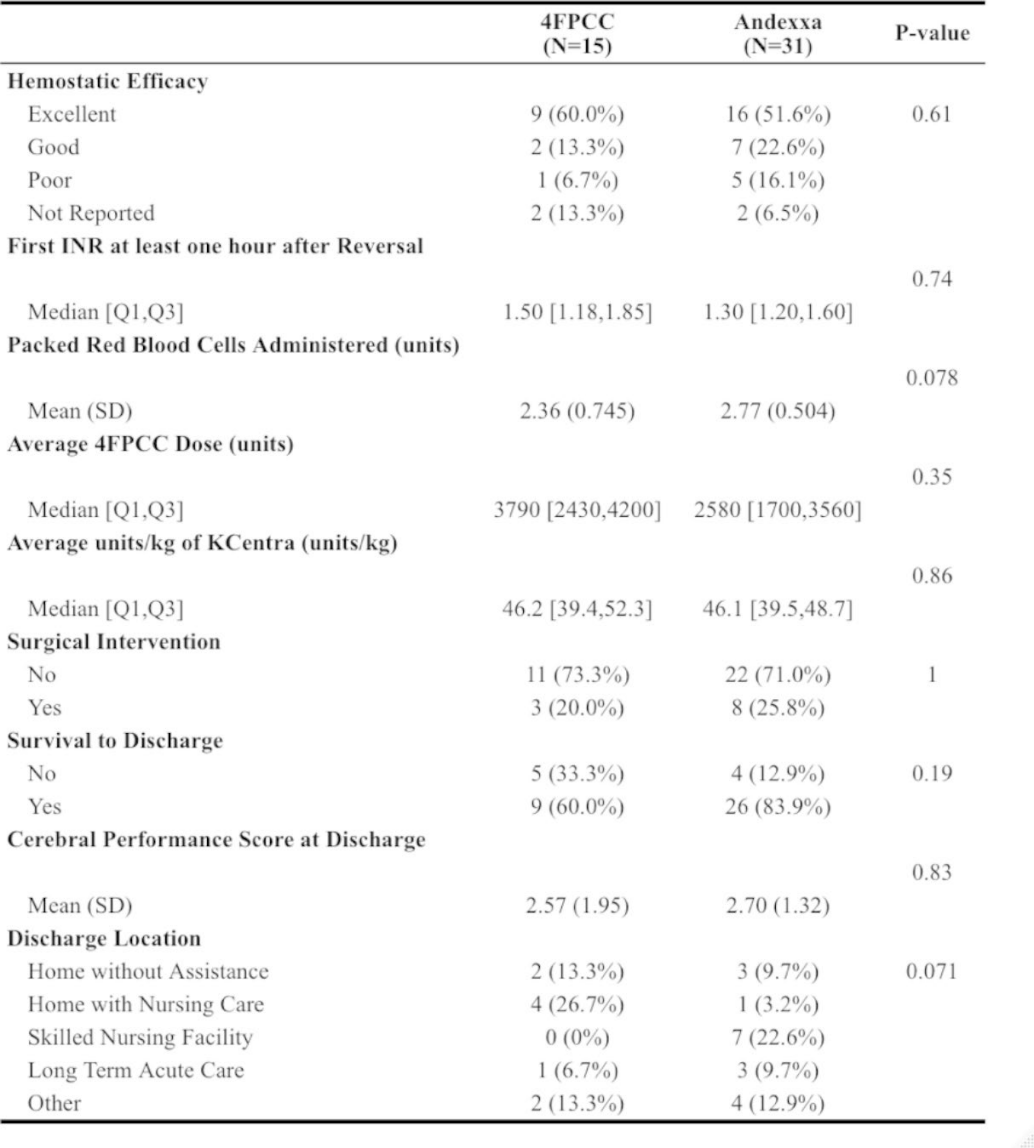
Primary and Secondary efficacy outcomes

**Table 6:**
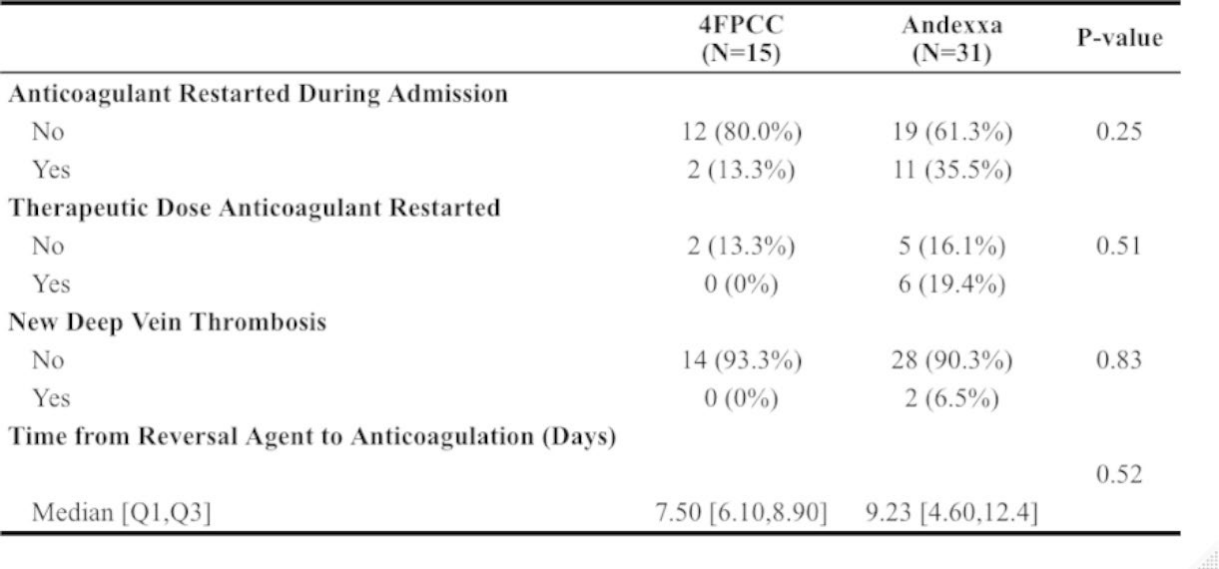
Safety Outcomes

**Fig. 1:**
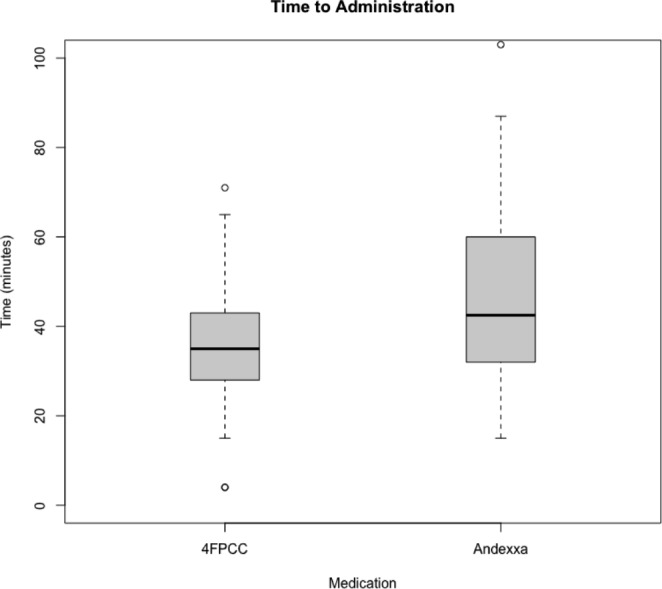
Boxplot of administration time

## Results

A total of 46 patients were included in this study. Of the 46 patients that presented with an ICH in the setting of recent DOAC administration, 15 received 4FPCC (32%) and 31 received andexanet alfa (68%). Both treatment groups had similar characteristics at baseline (Table 4) apart from age at time of administration (4FPCC, 69 years vs. andexanet alfa 77 years, *p* = 0.013). Most patients were male (4FPCC, 9 [60%] vs. andexanet alfa 18 [58.1%]), with similar weight (4FPCC 77.0 kg vs. andexanet alfa, 67 kg, *p* = 0.16) and serum creatinine at time of reversal (4FPCC 1.02 mg/dL vs. andexanet alfa 1.09 mg/dL, *p* = 0.66). There was no significant between group differences in the indication for DOAC administration, however, most patients were taking a DOAC for atrial fibrillation (4FPCC vs. andexanet alfa 9 [60%] vs. 21 [67.7%], *p* = 0.3). While between group differences were similar between DOACs; apixaban (4FPCC 8 [53.3%] vs. andexanet alfa 25 [80.6%], p = 0.11) and rivaroxaban (4FPCC 7 [46.7%] vs. andexanet alfa 6 [19.4%], p = 0.11) more patients were overall on apixaban. Further information regarding treatment characteristics is included in Table 4.

We found no significant difference when evaluating our primary outcome of hemostatic efficacy (Table 5). There was no difference in the proportion of patients with excellent (4FPCC 9 [60%] vs. andexanet alfa 16 [51.6%], p = 0.61), good (4FPCC 2 [13.3%] vs. andexanet alfa 7 [22.6%]), or poor (4FPCC 1 [6.7%] vs. andexanet alfa 5 [16.1%]) hemostasis after administration of each agent. Patients in the andexanet alfa group received a similar amount of blood products on average (4FPCC 2.36 units vs. andexanet alfa 2.77 units, p = 0.078). The number of patients with new thrombosis at 30 days was not statistically different between groups (4FPCC 0 [0%] vs. andexanet alfa 2 [6.5%], *p* = 0.83). There was no significant difference in survival to discharge, CPC score, or discharge location between groups. As shown in Fig. 1, there was also no difference in the time from order to administration for 4FPCC and andexanet alfa (4FPCC 35.3 min vs. andexanet alfa 47.8 min, p = 0.0538).

## Discussion

In this retrospective, single-center study, andexanet alfa and 4-factor prothrombin complex concentrate (4FPCC) were evaluated for the reversal of direct oral anticoagulants (DOACs) apixaban and rivaroxaban in patients with intracranial hemorrhage. No between group differences were found in the primary outcome of hemostatic efficacy or in secondary outcomes.

Hemostatic efficacy in our study is similar to previous studies evaluating the use of andexanet alfa and 4FPCC for DOAC-related bleeding reversal. A 2019 retrospective, single-center study by Ammar and colleagues evaluated outcomes of andexanet alfa and 4FPCC in adult patients admitted with a life-threatening traumatic or spontaneous intracranial hemorrhage in the setting of factor Xa inhibitor therapy [9]. Ammar and colleagues found there was no significant difference in neuroimaging at 6 and 24 h after administration of the reversal agent, degree of hemostasis based on hematoma volumes, number of patients with good outcomes upon discharge, or incidence of thrombotic events [9]. Other studies, while not powered to run statistical testing, showed similar trends of hemostasis among both andexanet alfa and 4FPCC treatment arms. One retrospective, observational study of patients presenting with ICH and anticoagulated with apixaban or rivaroxaban found there was 64.7% effective hemostasis in patients receiving andexanet alfa compared to 54.8% effective hemostasis in patients receiving 4FPCC [13]. A 2020 retrospective case series of patients 18 years of age or older on apixaban or rivaroxaban that received either andexanet alfa or 4FPCC for reversal of an ICH found there was 88.9% hemostatic efficacy in patients receiving andexanet alfa and 60% hemostatic efficacy in patients receiving 4FPCC, using the same criteria outlined in the ANEXXA-4 trial to define hemostatic efficacy [14, 15]. One important caveat to our data is that we did not meet statistical power by our calculation, which can lead to misinterpretation of the results by either type 1 or type 2 error. As such, it should be expressed that any perceived differences in the results only underscore the continued need for head to head prospective trial observation.

In secondary outcomes, we found no statistically significant difference in time from order to administration between 4FPCC and andexanet alfa. However, the difference may still be clinically meaningful. There are several reasons why this difference may occur at our institution. Dosing of 4FPCC is dependent on the actual factor IX content. When entering an order for 4FPCC the pharmacist must summate many different 500–1000-unit ranges of factor IX packages to get a total dose as near as possible to 50 units/kg. This results in an extended order entry and verification process when compared to andexanet alfa. Pharmacists with experience in its verification may be at the patient’s bedside and involved in care, slowing down the verification process. However, 4FPCC can be made at bedside while andexanet alfa must be compounded in a sterile IV room, which could lead to a faster administration time of 4FPCC when compared to andexanet alfa. Finally, andexanet alfa requires more manipulations with a bolus dose requiring 2–4 200 mg vials and 3–5 200 mg vials for the infusion dose. In summary, order entry and verification is more complicated for 4FPCC, however sterile manipulation and preparation is likely more complicated for andexanet alfa. Another important secondary outcome was discharge location as quality of life is an important consideration for survivors of ICH. While we found no statistical difference, there appear to be signals of differences between groups with respect to home discharge with services and skilled nursing facility discharge. While these decisions are multifactorial and may not be directly attributable to the agent of choice, this again underscores the need for protocolization and prospective study of the use of these agents.

In our study, there was not one agent that had a statistically higher rate of thrombosis. Although there were two events in the andexanet alfa group with one of these patients subsequently receiving 4FPCC after andexanet alfa, our study was not adequately powered to assess this outcome. A recent meta-analysis done by Gomez-Outes and colleagues and published in the Journal of American College of Cardiology found that the risk of thrombosis was higher with andexanet alfa than with 4FPCC (10.7% vs. 4.3%)[16]. This can possibly be explained by andexanet alfa’s mechanism of action. While andexanet prevents the action of factor Xa inhibitors, it also inactivates tissue factor pathway inhibitor (TFPI). This subsequently causes a transient increase in thrombin generation, which may potentially increase the risk of clotting [17].

There are several limitations to our study. First and foremost, this is a retrospective study, which limits selection of patients to those who received the intervention and doesn’t select for those patients who should have received a different treatment, or no treatment. Second, there is no institutional standardization for time to evaluation of the head CT after administration of reversal agent, leading to variability in the timing of follow-up which could potentially miss evolution of a bleed. Additionally, our center does not have 24-hour clinical pharmacist coverage. Complicating our data, there were three patients who received andexanet alfa and then 4FPCC, these patients were included in the andexanet alfa group, one of these patients developed a thrombus within 30 days. However, it is important to note that no patients who received 4FPCC first subsequently received andexanet alfa. It is also important to consider the FDA approval for each of these medications. Currently, 4FPCC is only FDA approved for reversal of non-vitamin K antagonist-related bleeding and is used off-label for bleeding related to direct oral anticoagulant use. Andexanet alfa is the only FDA-indicated reversal agent for DOAC-related bleeding and may be selected over 4FPCC due to liability concerns when using an off-label agent. Currently there is a registered clinical trial (NCT03661528) exploring andexanet alfa compared to standard of care, which usually includes 4FPCC. Ideally, this large randomized controlled clinical trial will provide more information on the role of andexanet alfa in the reversal of DOAC-related bleeding.

In our single center retrospective analysis of patients with DOAC-related intracranial hemorrhage, we found no difference in hemostatic efficacy between andexanet alfa and 4FPCC. At this time, clinicians should choose an agent based on individual patient presentation and resource availability. Further research is ongoing that will hopefully clarify the role of each agent in the management of DOAC-related intracranial hemorrhage.
